# Stone tools differences across three capuchin monkey populations: food’s physical properties, ecology, and culture

**DOI:** 10.1038/s41598-022-18661-3

**Published:** 2022-08-23

**Authors:** Tiago Falótico, Tatiane Valença, Michele P. Verderane, Mariana D. Fogaça

**Affiliations:** 1 School of Arts, Sciences and Humanities, University of São Paulo, Av. Arlindo Bettio, 1000, Prédio CMP1, Sala T6, São Paulo, SP 03828-000 Brazil; 2 Neotropical Primates Research Group, São Paulo, SP Brazil; 3 Institute of Psychology, University of São Paulo, São Paulo, SP Brazil; 4 University of Veterinary Medicine Vienna, Vienna, Austria

**Keywords:** Animal behaviour, Anthropology

## Abstract

Robust capuchin monkeys (*Sapajus*) are known for processing mechanically challenging foods, having morphological adaptations to do so. However, several populations go beyond body limitations by using stone tools to expand their food range. Those populations use stones in a variety of ways, goals, and with different frequencies. Stone tool size correlates with the food’s resistance within some populations. However, we have no detailed comparisons to identify if this correlation is the same across populations. This study described and compared stone raw material availability, food’s physical properties (hardness and elasticity), and stone tool weight in three populations of bearded capuchin monkeys (*Sapajus libidinosus*), including a newly described site (Chapada dos Veadeiros National Park, CVNP). The differences we observed regarding stone tool weight selection among sites were not correlated to the food’s physical properties we analyzed. Lithic resource availability could partly explain some differences in the stone tools used. However, the tool weight differences are larger than the raw material variance across sites, meaning some distinctions are possible behavioral traditions, such as the same fruit (*Hymenaea*) being processed with bigger than needed tools in CVNP than in the other two sites. Capuchin monkey behavioral variability in stone tool use can be caused by several interacting factors, from ecological to cultural.

## Introduction

Robust capuchin monkeys (genus *Sapajus*) are widely and long known for processing mechanically challenging foods^1–3^. The genus *Sapajus* exhibits adaptation in the skull and postcranial skeleton that should produce increased masticatory or postcranial forces when feeding, such as thick enamel, large mandibular corpus and symphysis, and a broad face^4,5^. However, there are several degrees of morphological variance between species that do not straightforwardly correlate with species diet differences. For example, *Sapajus apella* has a more robust skull and skeleton than *S. libidinosus,* but the latter has a diet with tougher food^3^. This counterintuitive fact may represent behavioral adaptation in opposition to morphological adaptation since *S. libidinosus*’ use stone tools to exploit the most challenging foods in their diet^6–12^. The same happens with yellow-breasted capuchins (*S. xanthosternos*), which also have a more gracile skull morphology than *S. apella*^5^, but can process hard food resources using tools^13^.

*Sapajus* is one of the few extant primate genera (together with *Pan*, *Homo, Macaca* and *Cebus*) to customarily use stone tools to process encapsulated food^14–16^. However, the use of stone tools is not the same in all populations, with variations in the material and dimensions of tools used and the food resources explored. Previous studies have suggested that the stone tools are chosen chiefly depending on the physical properties of the target food resource, and that was shown for populations of *S. libidinosus*, where heavier tools are used for harder food, as the stone tools used to process large palm nuts^17–19^. Additionally, chemical properties can also be a factor to influence the choice of a tool. For example, capuchins at Serra da Capivara choose larger tools to crack soft fresh cashew nuts, apparently, to protect themselves from the caustic liquid present at the fresh cashew nuts^20^. Differently, another population in which the same resource is explored without stone tools, uses a rubbing technique to avoid contact with the caustic liquid^21^. Therefore, information on ecology, lithic raw material availability, and behavior, including dietary ecology and food material properties, are needed to understand the evolutionary driving forces behind anatomical and cultural changes related to food processing.

The *S. libidinosus* population from Serra da Capivara National Park (SCaNP) is known to present, to date, the most diverse use of stone tools within the genus^9,19,22–24^. Besides using stone tools to crack open encapsulated plant resources, they also use stone tools to aid digging for food, and for other percussive behavior, such as stone on stone to obtain powder to ingest and anoint^19,25^. More recently, the population of *S. libidinosus* of Serra das Confusões National Park (SCoNP), 100 km from SCaNP, was described to use stone tools to process encased food, exploring different resources (*Buchenavia* nuts), or on different frequencies (*Attalea* nuts, *Hymenaea* pods), from other populations^6^. Another well-studied population of *S. libidinosus* is from Fazenda Boa Vista (FBV), where the monkeys appear to be specialized in cracking open hard resistant palm nuts^10,25^, although they also occasionally process less resistant food^26^, using lighter stone tools^10^. Other populations of *S. libidinosus* have been described to use stone tools to process encapsulated, hard-to-break food resources^7,8^. However, in no population, except for SCaNP, have capuchins been observed, so far, to habitually use stone tools for anything other than cracking encased food.

The relation between the food resource characteristics and stone tools has been studied to some extent in this species. Although the studies do not measure the resistance of the food, they argue that stone tools are most frequently used to process high resistant fruits, such as palm nuts^7,18,27–29^, and there is a correlation between hammerstone weight and the apparent higher resistance of the resources^6,18,19^. However, there is almost no information about actual values for resistance of the resources (but see^25^ for values of peak force to failure of palm nuts) and no comparison between capuchin populations concerning the physical properties of the processed food regarding the features of the stone tools used in each area.

Accessing the physical properties of the food can shed light on the tool choice by capuchins to process those foods and better understand the relation between resistance of food resources and tool features. The effectiveness of the forces produced when the tool contacts the target depends on the characteristics of the food and tool, but also the supporting substrate (anvil), and the interactions between them. There is a wide range of characteristics that could influence the tool choice, such as surface material, size and shape; and target mechanical properties (e.g., size, elastic modulus and toughness). Once the food is hit, the resistance to fragmentation is defined by the internal mechanical properties. The size, shape, and physical properties of the tool interact with the same properties of the food, and we can infer that the food breakdown is optimized to the animals' needs, saving energy. To measure the internal mechanical properties, tests record the force applied to the object and the deformation that it produces. The deformation can be registered as a force–displacement graphic. Hardness is the resistance to the initial loading force; in other words, the bigger the hardness value, the higher the force necessary to initiate a fracture. Elastic modulus corresponds to the material’s rigidity and can also be used to estimate food resistance to the beginning of a fracture; thus, less elastic material (lower values of the elastic modulus) would be more rigid^30^.

**Figure 1:**
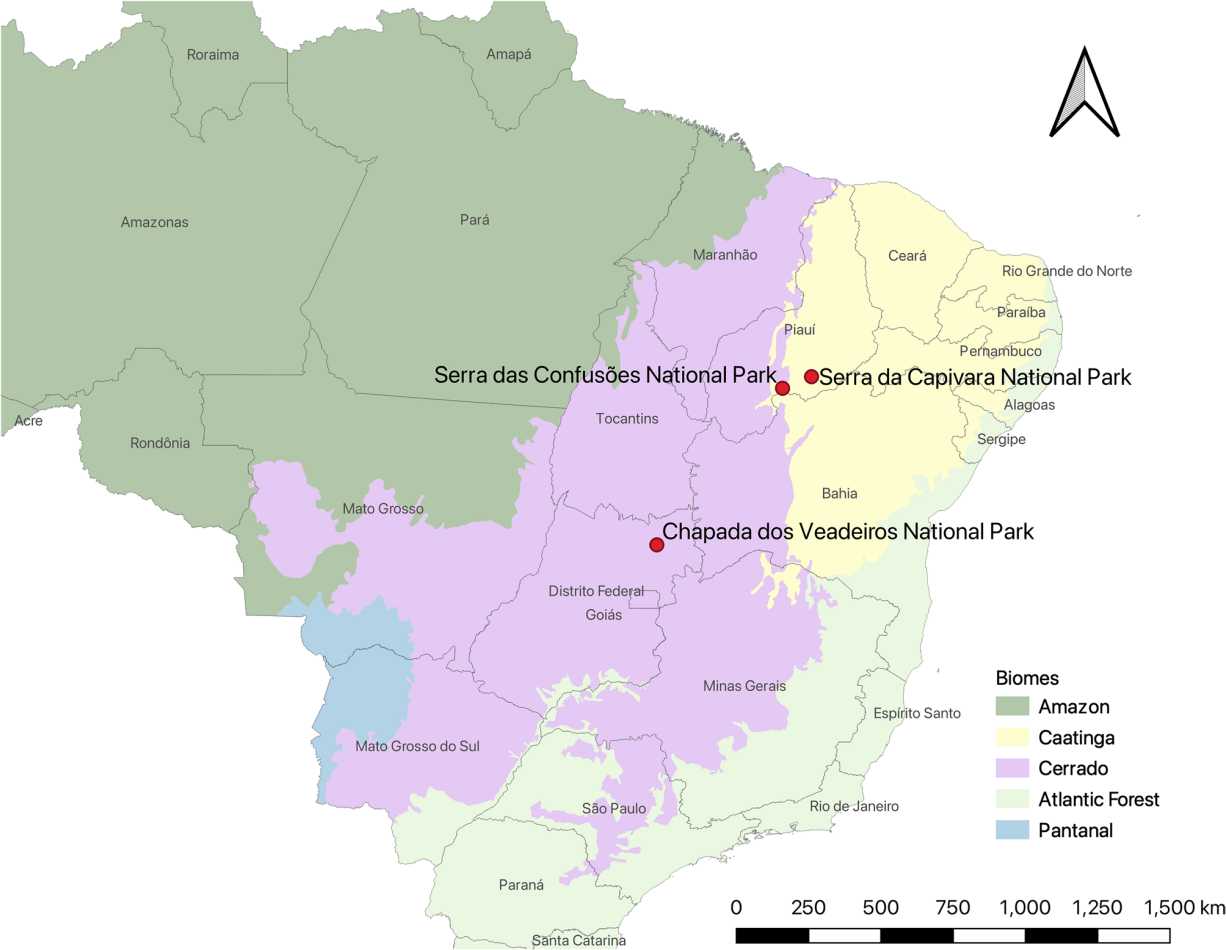
Map of Brazil with the locations of the study sites on this work (red dots). Biomes are indicated by color (see legend). Map created by Tiago Falótico, using QGIS 3.16 (https://www.qgis.org) and biomes data from IBGE, Instituto Brasileiro de Geografia e Estatística (https://ibge.gov.br).

In this study, we described and compared lithic raw material availability, food resources physical properties, and features of the stone tools used (hammer and anvil) of three populations of bearded capuchin monkeys (*S. libidinosus*) that use stone tools to process food resources (Fig. 1), to test the following hypotheses:

1. Capuchin monkeys in each population select stones of adequate weight to process the food resources present in their living area.
  Prediction 1a—Hammerstone weight will be correlated to food resource hardness and elasticity, heavier stones to harder and less elastic resources.
  Prediction 1b—Anvil dimension will be positively correlated to food resource length.
2. The same food resources explored by capuchins in different populations have similar physical properties.
  Prediction 2—The hardness and elasticity of the same food resources in different areas will have similar values.

## Results

Stone tools dimensions of the three study sites are presented in Table 1. Stone tools at CVNP were statistically heaviest than the other sites (see section “Comparison between sites” for the statistical results). The stone tools were composed of quartzite, sandstone, and limonite, with different frequencies (χ^2^ = 212.42, n = 486, df = 4, p < 0.001) at each site (Table 2).

**Table 1:** Pounding stone tools and anvil general dimension for each site, presenting average, standard deviation, and range values.

	N	Weight (g) mean ± SD, range	Length (mm) mean ± SD, range	Width (mm) mean ± SD, range	Thickness (mm) mean ± SD, range
**CVNP**					
Anvil	119	–	499 ± 270.9, 57–1750	315.3 ± 176.5, 90–870	162.5 ± 138.6, 20–900
Hammer	140	1672.2 ± 1050.6, 266–5700	152.8 ± 41.6, 71–285	102.8 ± 26.9, 52–195	74.5 ± 63.7, 20–790
**SCaNP**					
Hammer	542	202.5 ± 209.1, 18–1900	70.1 ± 23.6, 31.5–210	52.2 ± 23.1, 24–427	37 ± 12, 14–84
**SCoNP**					
Anvil	57	–	449.5 ± 256.9, 88–1410	309.4 ± 183.7, 48–970	–
Hammer	69	316 ± 254.4, 31.4–1409	96.6 ± 30.7, 44.7–210	56.8 ± 15.7, 29.8–95.3	33.6 ± 11.4, 12.5–66.8

Sources: *CVNP*—Chapada dos Veadeiros National Park (this work), *SCaNP*—Serra da Capivara National Park^19,31^, *SCoNP*—Serra das Confusões National Park^6^. Anvil data for SCaNP is not presented because the previous work on the site did not register that measurement.

**Table 2:** Stone tools characteristics in each site.

Site	N	Quartz	Sandstone	Limonite	Conglomerate
CVNP	180	58.9%	21.7%	19.4%	0
SCaNP	237	89.1%	8.8%	1.3%	0.8%
SCoNP	69	0	40.6%	59.4%	0

The lithic raw material characteristics available for each area are presented in Table 3, and were significantly different across the sites (χ^2^ = 277.25, n = 577, df = 4, p < 0.001).

**Table 3:** Lithic raw material characteristics in each site, from plot samples.

	N	Weight (g) mean ± SD, range	Length (cm) mean ± SD, range	Density (stone/ha)	% material
**CVNP**	216	216.3 ± 574.1, 6.4–6450	6.5 ± 3.93, 3–24.5	480	
Quartz	97	297.6 ± 770.1, 7.4–6450	7.0 ± 4.3, 3–24.5	215.5	44.9%
Sandstone	17	86.2 ± 158.5, 8.2–672	5.1 ± 2.39, 3.3–12.9	37.8	7.9%
Limonite	100	154.9 ± 346.7, 6.4–2250	6.2 ± 3.71, 3–24.5	222.2	46.3%
Conglomerate	2	447.5 ± 218.5, 293–602	12.1 ± 2.24, 10–14	4.4	0.9%
**SCaNP**	188	113.5 ± 185.1, 8.8–1120	5.5 ± 2.83, 3–22	417.84	
Quartz	145	104.1 ± 180.1, 8.8–1120	5.0 ± 2.11, 3–14.6	322.2	77.1%
Sandstone	43	145.0 ± 199.8, 8.9–840	7.2 ± 4.08, 3.2–22	95.5	22.9%
**SCoNP**	176	149.8 ± 281.1, 3.1–1950	6.6 ± 3.55, 3–25	391.1	
Sandstone	70	164.3 ± 277, 3.1–1399	6.8 ± 3.63, 3–16.7	155.5	39.8%
Limonite	105	140.8 ± 285.9, 6.8–1950	6.4 ± 3.5, 3–25	233.3	59.7%
Resin	1	85.4	10.3	2.2	0.6%

Only stones with more than 3 cm were considered in this sampling.

The primary food resource found at the anvils during the mapping at CVNP was *Attalea* palm nut (91%); *Hymenaea* remains were found in only 3% of the mapped anvils. At SCaNP the main resources for nut-cracking observed were cashew nuts (58%) *Manihot* (13%), *Cordia* fruits (8%), and *Hymenaea* (6%)^19,31^. Finally, at SCoNP, the main resources found in the anvils were *Buchenavia* fruits (38%), *Manihot* (31%), *Hymenaea* (19%), and *Attalea maripa* nuts (11%); the remaining 1% was not identified^6^.

**Figure 2:**
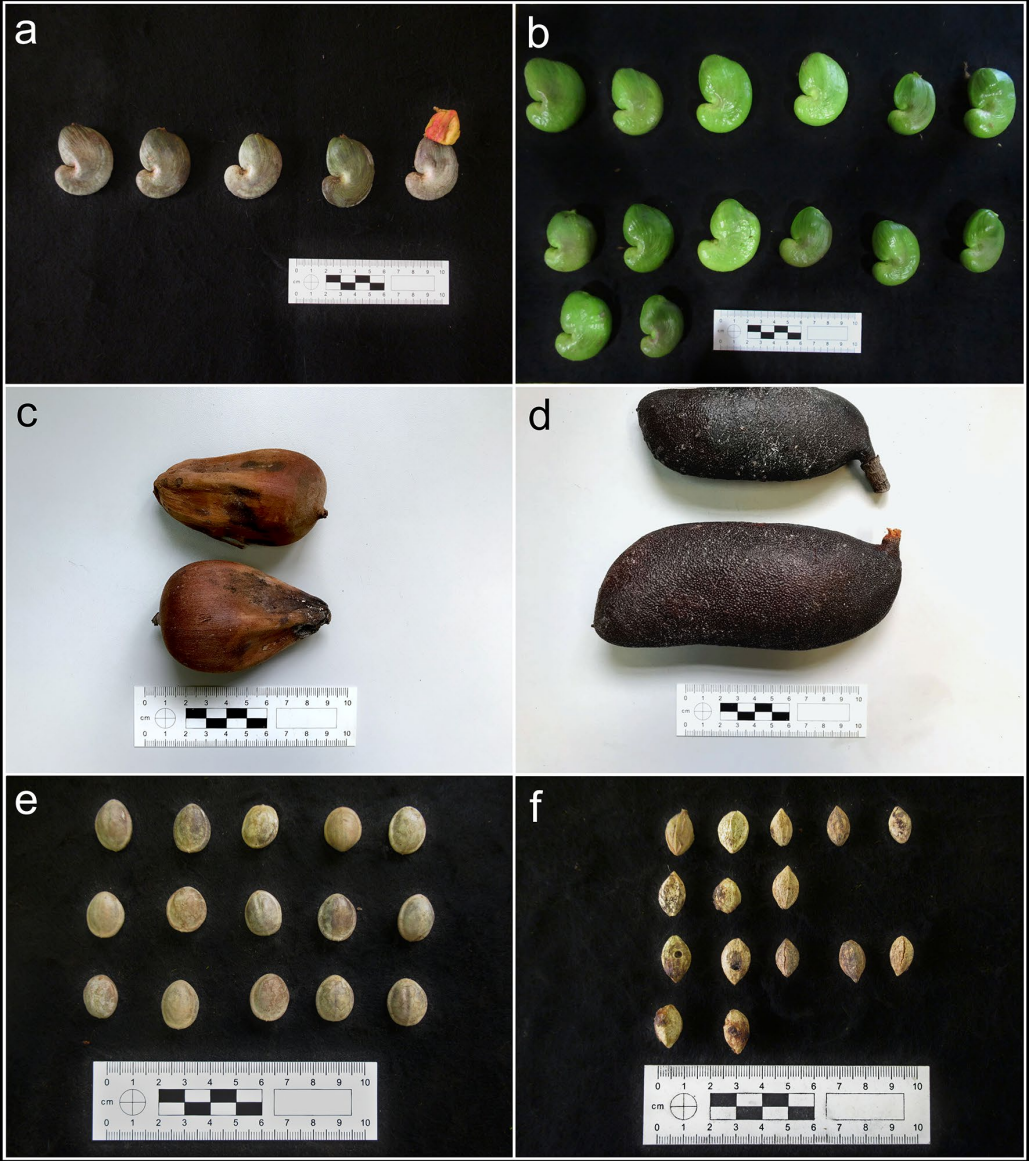
Food resources sampled. (**a**) Dry cashew nut, *Anacardium*; (**b**) fresh cashew nut, *Anacardium*; (**c**) palm nut, *Attalea*; (**d**) jatobá pod, *Hymenaea*; (**e**) *Manihot* seed; (**f**) Mirindiba, *Buchenavia grandis*.

The physical properties test results (Indentation Hardness and Reduced Elastic Modulus) on the resources sampled in each area (Fig. 2), plus the mean weight of stone tools used to process each kind of resource, are presented in Table 4.

**Table 4:** Physical properties (indentation hardness and elastic modulus) of the food resources processed with stone tools in each area and the average stone tool weight the capuchin monkeys use to process each resource type.

Resource	N	Indentation hardness (MPa) mean ± SD	Reduced Elastic Modulus (GPa) mean ± SD	Length (mm) Mean ± SD	Avg stone tool weight (g)
**Anacardium**	33	3.40 ± 5.53	0.0642 ± 0.092	37.79 ± 8.05	195.8 (n = 34)
Dry nut	11	8.56 ± 7.25	0.1559 ± 0.113	32.55 ± 2.32	
SCaNP	5	13.72 ± 7.68	0.2472 ± 0.096	32.28 ± 1.1	186.9 (n = 12)
SCoNP	6	4.27 ± 3.03	0.0799 ± 0.052	32.78 ± 3.11	n.a
Fresh nut	22	0.81 ± 0.78	0.0184 ± 0.044	40.41 ± 8.65	
SCaNP	9	1.28 ± 1.03	0.0206 ± 0.017	49.78 ± 3.92	204.8 (n = 22)
SCoNP	13	0.49 ± 0.28	0.0168 ± 0.044	33.92 ± 3.04	n.a
**Attalea**					
CVNP	21	104.68 ± 91.06	3.7363 ± 3.207	63.33 ± 5.2	1723.4 (n = 134)
**Hymenaea**^**#**^	21	36.25 ± 16.72	0.8845 ± 0.457	–	537.9 (n = 44)
CVNP	5	24.08 ± 4.56	0.5550 ± 0.093	–	1011.2 (n = 4)
SCaNP*	7	35.12 ± 11.54	1.1014 ± 0.513	–	249.2 (n = 14)
SCoNP	9	42.54 ± 20.9	0.8622 ± 0.446	–	353.3 (n = 26)
**Manihot**	72	118.24 ± 73.29	2.115 ± 2.379	16.42 ± 1.27	195.3 (n = 50)
SCaNP	43	83.58 ± 47.83	2.1973 ± 2.954	15.73 ± 1.07	169.3 (n = 30)
SCoNP	29	169.64 ± 74.88	1.9931 ± 1.113	17.30 ± 0.93	221.4 (n = 20)
**Buchenavia**					
SCoNP	10	51.52 ± 32.04	0.7732 ± 0.562	16.09 ± 1.54	342 (n = 18)

Some values are not available (n.a.) because the monkeys were not observed to process the resource with stone tools in that population.

*Data on stone tool weight for *Hymenaea* in SCaNP from^31^.

^#^The *Hymenaea* species at SCoNP is *H. martiana*, and in SCaNP is *H. courbaril*. The species sampled at CVNP is unknown, as there are two similar species in the area, *H. courbaril* and *H. stignocarpa*^32^. We compared them at the genus level in this work. The length value is not shown for *Hymenaea* because the samples were often fragmented, not allowing the measure of the original length.

There was no correlation between the average values of stone tool weight and resource hardness (Spearman, S = 16, p = 0.297), or tool weight and resource elasticity (Spearman, S = 16, p = 0.297). This test was performed with the average values because the food sample could not be linked to each stone tool's weight values, as they were collected separately.

### Comparison within populations

**Figure 3:**
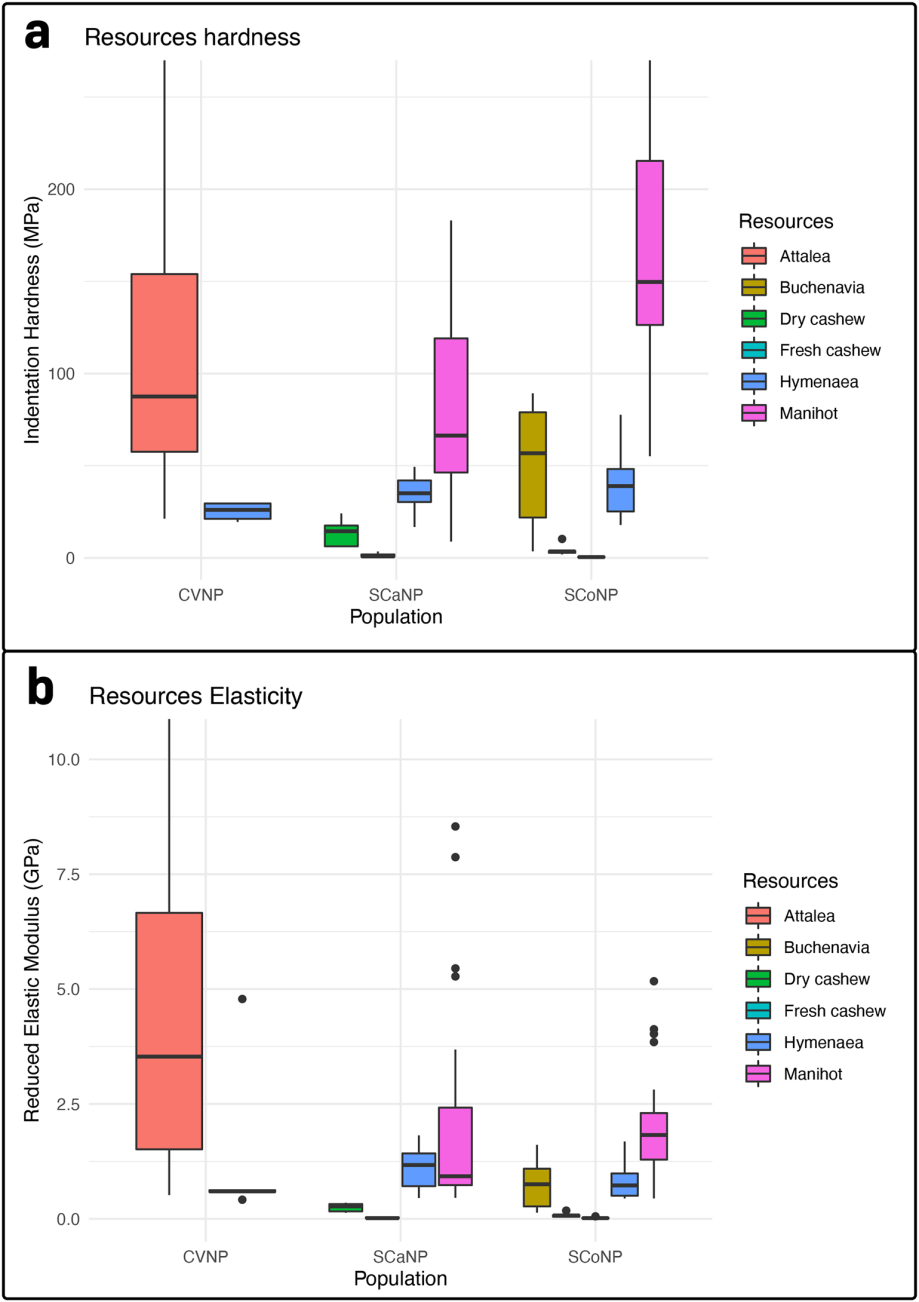
Physical properties of the food resources processed with stone tools in each study site. (**a**) Food resources hardness per study site. (**b**) Food resources elasticity per study site.

**Figure 4:**
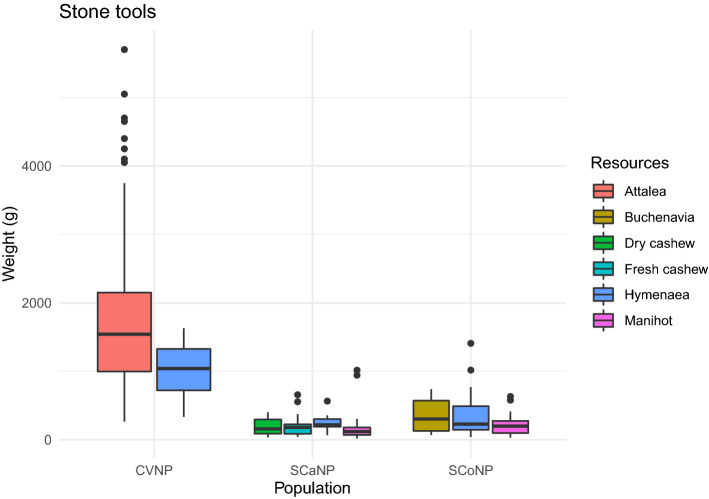
Hammerstone tool weight to process each resource at each site.

The resource physical properties and stone tools used to process those items in each population are summarized in Figs. 3 and 4.

In CVNP, the two resources (*Hymenaea* pods and *Attalea* palm nuts) analyzed were statistically different in elasticity (Mann–Whitney, W = 100, n = 29, p = 0.019), and not significant for hardness (Mann–Whitney, W = 93, n = 29, p = 0.058). Cohen's d for the elasticity indicates that the effect size was large (d = 0.88). Still, the hammer stone weight was not significantly different (Mann–Whitney, W = 378.5, n = 138, p = 0.163). However, we take this result cautiously because only four tool sites used to process *Hymenaea* were recorded.

In SCaNP, the resources (*Hymenaea* pods, *Manihot* seeds, and *Anacardium* cashew nuts) had significant differences in hardness, elasticity, and weight of the stone tools associated (Kruskal–Wallis, χ^2^ = 37.348, n = 64, df = 3, p < 0.001; χ^2^ = 33.005, n = 64, df = 3, p < 0.001; χ^2^ = 8.533, n = 79, df = 3, p = 0.036). However, the weight of the stones was only significantly different in one pairwise comparison, revealing that the only difference was between hammer stones to process *Hymenaea* pods and *Manihot* seeds (Mann–Whitney, W = − 2.948, n = 79, p = 0.003), the latter processed with lighter stone tools.

Finally, in SCoNP, the resources (*Buchenavia* seeds, *Hymenaea* pods, *Manihot* seeds, and *Anacardium* cashew nuts) had significant differences in hardness and elasticity (Kruskal–Wallis, χ^2^ = 55.493, n = 66, df = 4, p < 0.001; χ^2^ = 50.203, n = 67, df = 4, p < 0.001). Pairwise comparison showed that the only resources that did not significantly differ in the tested properties were *Buchenavia* seeds and *Hymenaea* pods (hardness, Mann–Whitney, W = − 0.816, n = 66, p = 0.447; elasticity, Mann–Whitney, Z = − 0.568, n = 67, p = 0.604). The hammerstones associated with the *Buchenavia* seeds, *Hymenaea* pods, and *Manihot* seeds in SCoNP did not significantly differ in weight (Kruskal–Wallis, χ^2^ = 3.364, n = 66, df = 2, p = 0.186).

Regarding SCoNP anvils, we found no differences between the length of the anvils used to process the food resources with hammerstones (Kruskal–Wallis, χ^2^ = 1.516, n = 70, df = 3, p = 0.679).

### Comparison between populations

To analyze the differences between populations, we compared the resources present in more than one site and the stone tools used to process those items.

*Attalea* palm nuts, *Buchenavia* seeds, and the associated stone tools were not quantitatively evaluated because they are present in only one of the study sites.

Overall, the stone tools analyzed were significantly different between populations (Kruskal–Wallis, χ^2^ = 196.73, df = 2, n = 278, p < 0.001). CVNP had the heaviest tools compared to the other places (Wilcoxon pairwise comparison with Bonferroni correction, p < 0.001), and SCoNP stone tools were heaviest than the SCaNP ones (Wilcoxon pairwise comparison with Bonferroni correction, p = 0.007). The effect size was large for the CVNP with the other populations (Cohen’s d, CVNP/SCaNP, d = 1.77; CVNP/SCoNP, d = 1.58) and medium for the relation between SCaNP and SCoNP (Cohen’s d = − 0.50).

The stones available in CVNP were 1.4 and 1.9 times heavier than the ones in SCoNP and SCaNP respectively (Table 3). However, the stone tools in CVNP were 5 to 8 times heavier compared to the same sites, suggesting that the heavier stone availability is not the only factor that causes the tool selection in CVNP and that an active choice for heavier tools is happening in that site.

Fresh cashew nuts sampled in SCaNP and SCoNP were similar in elasticity (Mann–Whitney, W = 62, n = 22, p = 0.845) but had significantly different values for hardness (Mann–Whitney, W = 92, n = 22, p = 0.025). The effect size in this last case was large (Cohen’s d = 1.15). As for dry cashew, we found significant differences in both physical properties (Mann–Whitney, W = 28, n = 11, p = 0.017; Mann–Whitney, W = 28, n = 11, p = 0.018), with SCaNP nuts being harder and less elastic (effect size were both large, Cohen’s d = 1.68 and d = 2.2, respectively). We could not compare stone tools for cashews because SCoNP monkeys had not been observed to explore this resource, with or without tools^6,33^.

Manihot seeds from SCaNP and SCoNP differed significantly regarding physical properties (Mann–Whitney, W = 191, n = 71, p < 0.001; Mann–Whitney, W = 447, n = 72, p = 0.043), SCoNP seeds were harder and less elastic. The effect size for hardness was large (Cohen’s d = − 1.40), but for elasticity was small (Cohen’s d = 0.08). The stones used to process those resources were not significantly different, but there was a trend (Mann–Whitney, W = 213, n = 50, p = 0.087).

The *Hymenaea* pods were the only resource analyzed in all three sites. We found no differences in physical properties between sites (Hardness, Kruskal–Wallis, χ^2^ = 0.978, df = 2, n = 21, p = 0.613; Elasticity, χ^2^ = 1.360, df = 2, n = 21, p = 0.507). Even though some of those resources are possibly from different species, the properties are similar. On the other hand, the hammerstone tools were significantly different in weight (Kruskal–Wallis, χ^2^ = 7.407, df = 2, n = 46, p = 0.025). Wilcoxon pairwise comparison showed the difference was between the stones from CVNP and the other sites (SCaNP, p = 0.008; SCoNP, p = 0.032). The effect size was large for both comparisons (Cohen’s d, CVNP/SCaNP, d = 2.9; CVNP/SCoNP, d = 1.91). The weight of hammerstone tools to process *Hymenaea* pods from SCaNP and SCoNP were not different (p = 1).

## Discussion

This work compared wild capuchin monkeys from three areas, describing the stone tool use in a new *S. libidinosus* population and comparing the resources processed within and between sites. We also described the physical properties of the resources and the stone tools used to process those foods and compared the data between the three sites and the literature. The differences we observed regarding stone tool use among sites, such as differences in stone tool weight to process the same resource (*Hymenaea*) in one of the studied populations, and the lack of differences within populations regarding the stone tools could be partially explained by ecological factors, such as raw material and resource availability. However, other differences appear to be more related to behavioral traditions.

**Figure 5:**
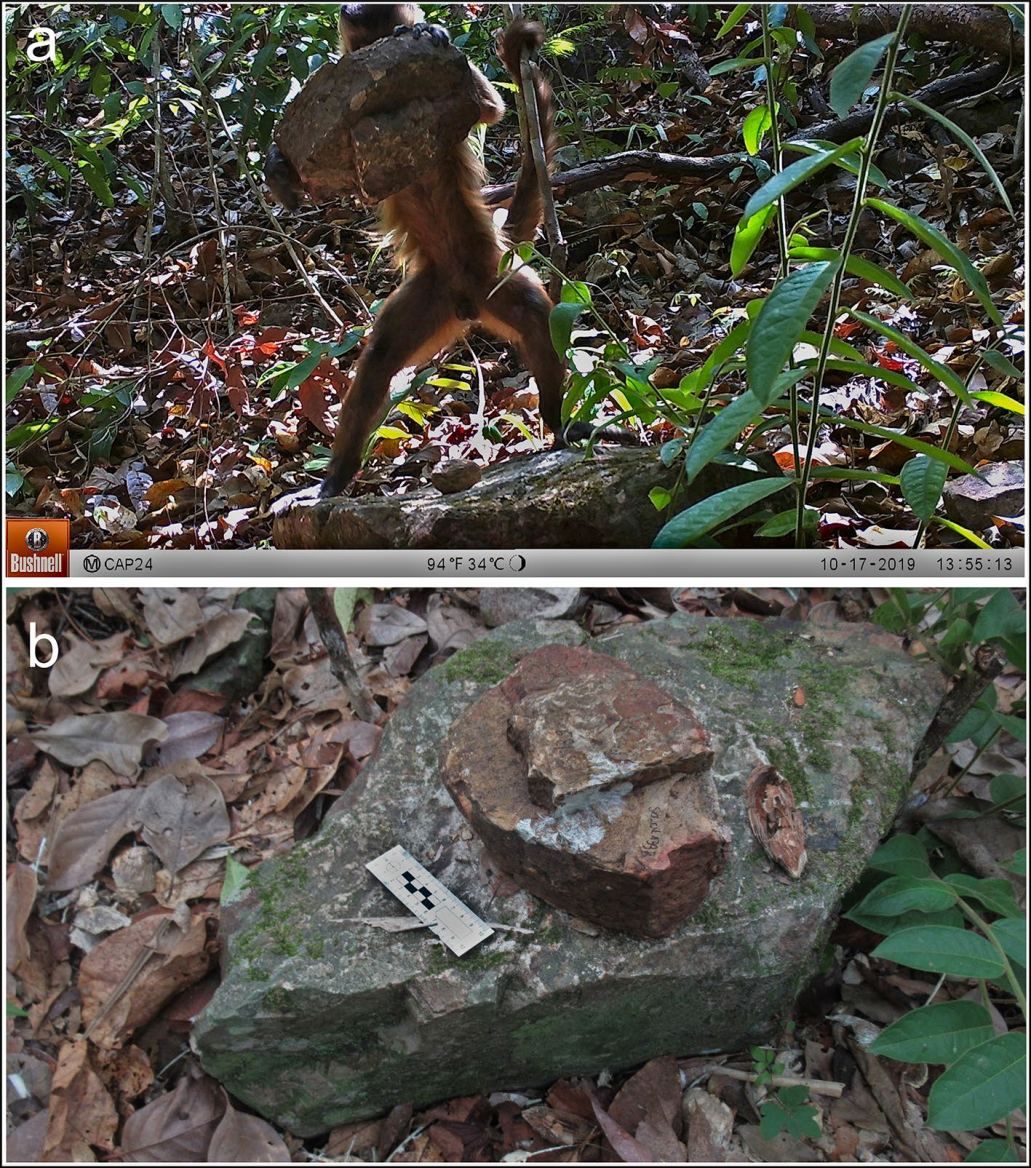
(**a**) Capuchin monkey from CVNP recorded on camera-trap using a 4650 g stone tool to crack open an *Attalea* palm nut. (**b**) The stone tool used. 10 cm scale.

The new capuchin population described at CVNP, in a typical tropical savannah (Cerrado), presents a similar stone tool use pattern observed in other Cerrado populations. Most of the stone tools at CVNP were found to be used for processing high resistant palm nut (*Attalea*), similar to those processed in other savannah environments (e.g., *Attalea barreirensis*, *Attalea speciosa*)^7,13,17^. However, the weight of the tools and the physical properties of the food processed at this site was bigger than the other two populations analyzed (and also from the literature). Stone tools in CVNP had an average weight of 1672 g. The heaviest stone tool registered in our mapping was 5700 g (Table 1). In latter camera-trap surveillance (data not presented here, see Supplementary Video 1 for examples), we recorded monkeys effectively using a 4650 g stone tool (Fig. 5), an astonishing weight to be lifted by monkeys with a body mass average of 3500 g for adult males and 2100 g for adult females^34^. At the other two sites (SCaNP and SCoNP), the resources explored had, overall, lower hardness values, and the stone tools used to pound food resources were lighter, as expected based on SCaNP and SCoNP previous results^6,19^, as well from other *S. libidinosus* populations (Fazenda Boa Vista and Luis Gomes) where the monkeys choose stones according to the resistance of the resource processed^10,18^. CVNP has a relatively high density (215 stones/ha) of quartzite pebbles (the best raw material for stone tools) (Table 3), making it much more likely for the monkeys to encounter and use it than other stones available. Furthermore, the quartzite pebbles in this site are on average larger than the stones available at the other sites, maybe allowing CVNP´s monkeys to more often use heavy stones. Thus, this environmental difference could partially explain the use of larger tools for the CVNP population. However, the tools used are much heavier than the average stones available, suggesting an active choice for bigger stones.

In SCaNP, the highest availability of the best raw material for stone tools, quartzite, compared to the other sites (322 quartzite stones/ha, 50% higher than CVNP) could explain the more diversity of stone tool use observed in that area than any other site^6,19,23,35^, as the availability of this material would allow more interaction with those, and, over time, more innovation^35^. However, we probably do not have yet a complete picture of the repertoire of stone tool use at CVNP to thoroughly compare those sites and better understand the mechanism underlying tradition on the use of tools.

One of the resources explored with stone tools in CVNP is jatobá pods (*Hymenaea* sp.), although much less frequently than the palm nuts, at least in the study areas we mapped. The stone tools used for *Hymenaea* were lighter on average than the ones used to process the harder palm nuts, although not statistically significant (there was, however, a trend). This result must be taken with caution because of the small sample of *Hymenaea* tools.

*Hymanaea* pods were the only resource present in all three populations compared here. Our results showed that the physical properties of the pods were not significantly different across sites, meaning the challenge to open the pods would be similar in all three areas. However, the weight of the stone tools used in each population was different. CVNP monkeys used much larger stones to process the pods than the monkeys from the other two populations. One explanation could be the availability of the potential material to be used as tool. As mentioned before, CVNP has the larger/heavier raw stone material availability (Table 3). An alternative explanation would be related to the other resources explored. At the CVNP area, the monkeys explore, at the same nut-cracking sites, and with higher frequency, a much harder food (*Attalea* nuts), which could bias the tools selection to larger stones because of those more resistant palm nuts.

To better understand the tool use repertoire at CVNP, we still need to directly observe the monkeys or perform experimental tests to check the existence (or not) of tool use observed in other populations (e.g., probe tools, digging stones). The use of camera-traps recording nut-cracking sites (analysis ongoing) will also allow the observation of the stone tool use, its efficiency, and variation.

The first prediction of hypothesis 1 was not supported in any of the analyzed populations. At the populational level, heavier stone tools were not correlated to harder or less elastic food. In the case of CVNP, we had a small sample for stone tools used for *Hymenaea*, and both resources analyzed were found close to each other, so the stone selection in that area could have been biased to the process of the harder *Attalea* nuts. In the case of SCoNP, stone tools' weights did not differ between tools used for *Hymenaea* and *Manihot* seeds. Two things could explain this difference. First, in SCoNP, the lithic raw material is scarcer than in CVNP and SCaNP, restricting the selection of tools. Second, although small (Avg length, 16.42 mm), *Manihot* seed showed surprisingly higher hardness values, perhaps requiring a similar stone tool to the ones used to open the larger *Hymenaea*. Considering only the hardness value, we would expect that the monkeys would use heavier stones to process *Manihot.* However, in SCaNP, the stones used to crack *Manihot* were lighter than *Hymenaea*. One possible explanation is that although *Hymenaea* is much larger, hardness does not directly scale with size, as there is a combination of internal structures and material factors. Considering the structure of the food resources, it could be helpful in future studies to clarify the stone dimension and food physical properties.

The second prediction for H1 was also not supported. The anvil length did not vary by resource size. Our explanation is that an adequate anvil can be successfully used to process most resources. So, not being a limiting factor that needs to be different for most resources, e.g., the same anvil used to process an *Attalea* nut could be potentially used for *Hymenaea* pods, particularly when both species occur near each other. Even when a small target, such as *Manihot*, is being processed, the choice of an anvil could be driven by the larger resource in the area (rule of thumb to choose larger anvils), as this larger anvil can be used for any resource and there are no energetic costs, as it is not transported. A factor that should be analyzed in the future is the inclination of the anvil or the presence of pits, as some resources are rounder and more prone to roll from a tilted anvil^17^, meaning this factor could be more critical for anvil selection depending on the type of resource to be placed on the anvil.

Our results show a more complex association between food physical properties and tool selection than previous studies^10,18,19^. The data presented here, although limited by the small sampling and non-parametric univariate analysis, suggest that variables probably interact, such as stone availability and food hardness, and it reflects on tool selection. Our results suggest an approach to the tool choice considering food hardness, size, and stones availability in the environment. Other factors such the individual size, strength, age, and even position in the hierarchy could also influence tool choice and were not taken into account in this work. Future work testing the relationships between physical properties and tool selection in other taxa will improve the generalizability of these results, as well including other factors in the analysis.

Hypothesis 2 was supported for *Hymenaea* pods and fresh cashews but not for dry cashews and manihot seeds.

*Hymenaea* pods and fresh cashews did not differ in the physical properties between sites, even though the *Hymenaea* in each place are from different species. That allows an interesting comparison between the use of stone tools to open this resource, as it is processed with stones of different weight even being similar regarding physical properties. The same is true, in part, for the green cashew nuts, although in this case, the chemical characteristics could also be a factor of influence^20^.

We found differences in hardness and elasticity between the study sites for *Manihot* and dry cashews. The difference between physical properties for the same species in different sites can be related to the time lying on the ground. Cashew nuts can lay on the floor for a long time (days or even months) before being picked by the monkeys (T.F. personal observation), and our sampling reflects that, as we collected a mix of dry nuts from the ground and directly from the tree. Therefore, we would expect the loss of water content to turn the tissues less elastic and harder^30^. The influence of the effects of the environment on the physical properties could also explain the difference found for *Manihot* seed; even if similar to the naked eye, we may have sampled seeds in different stages of desiccation.

Robust capuchin monkeys are known to explore mechanically tough food^2,3^. Capuchins also have features that may facilitate the production of large muscle and bite forces without compromising gape and so can exploit mechanically challenging foods at relatively large jaw gapes^36,37^. However, food such *Attalea* and *Hymenaea* are larger/bigger than the jaw gape of the monkeys and could only be opened by hand or using tools, and that appears to be the case. The monkeys at the analyzed populations (and other previously studied) have shown that the use of stone tools provides access to hard-encased food that would not be accessible otherwise^6,7,9,19,38^. Even for smaller food targets, e.g., *Manihot* seeds and cashew nuts, the efficiency of using tools to process those targets could be higher, especially if the population already has the tool use behavior in their repertoire because of the more challenging targets that obligatory need stone tools to be accessed, as appears to be the case of the populations in this study.

The differences we observed regarding stone tool use between the newly described capuchin population (CVNP) and the previously studied ones could be partially explained by ecological factors, such as the availability of lithic material and food resources. However, the differences in the availability of lithic material are not big enough to fully explain the behavioral variances. Our results show that capuchins choose, among the raw material available in the environment, suitable stone tools to open the food. Although the presence of other food resources can bias the tool choice, as the harder *Attalea* nuts presence appears to do at CVNP regarding *Hymenaea* stone tools. Moreover, some differences are more subtle, such as the cashew nut processing. Even though cashew nuts were available, SCoNP monkeys do not eat cashew nuts, with or without stone tools^33^. Since the absence of consumption of this food resource in SCoNP is neither because of availability nor impossibility of access, one could argue that this difference is a cultural behavior. The same can be said about the bigger stones used to process *Hymeanea* at CVNP^20^. Animal culture can be defined as all that is learned from others and is repeatedly transmitted in this way, forming traditions that may be inherited by successive generations^39^. The social learning of stone tools by capuchin monkeys has been shown several times^27,40,41^, but we still need to understand the ecological factors that also influence this stone tool’s cultural variance. Identifying behavioral differences across populations and the factors that lead to this variance is the first step to better understanding capuchin cultures.

## Methods

### Study sites

Serra da Capivara National Park (SCaNP, − 8.833239, − 42.552377) and Serra das Confusões National Park (SCoNP, − 9.213317, − 43.498371) are located in the south of Piauí state (Fig. 1), Brazil. Both parks have Caatinga (thornbush savannah) as the predominant biome and semiarid as the prevailing climate. Still, the west area of SCoNP is an ecotone with the biome Cerrado (tropical savannah). The vegetation is xerophytic, especially at higher elevations, but there are patches of deciduous forest in the humid valleys surrounded by high cliffs.

SCaNP has an area of 130,000 ha, with an average temperature of 28 °C (range 10–47 °C) and a mean annual rainfall of 689 mm. This region is composed of Serra Grande formation, which has sandstones and conglomerates, and Canindé Group, which has laminated shale, sandstones, tempestites, mudstones, cobbles, and siltstones (for more details, see^31,42^).

SCoNP has 823,435 ha of area, in which the average temperature is 28 °C (range 12–45 °C), and the mean annual rainfall is 650 mm. There are also more humid areas (Cerrado) in the east and north of SCoNP and some intermittent rivers in the valleys. This park presents three geological formations: Cabeça, which has hard sandstones; Pimenteiras, having red shale, sandstones, and siltstones; and Serra Grande, which has conglomeratic sandstone and quartz pebbles (for more details, see^6^).

Chapada dos Veadeiros National Park (CVNP, − 14.093696, − 47.373306) was created in 1961 in the northeast of Goiás State (Fig. 1), in a central region of Brazil. This site has an area of 240,611 ha characterized by Cerrado biome and a tropical savanna climate. It has annual rainfall ranging from 1500 to 1750 mm, most of it occurring during the wet season (November to March), and average temperatures ranging from 20 to 27 °C^32^. The region encompasses waterfalls and three rivers descending from high plateaus of altitudes up to 1676 m, erosional scraps, and intra-plateau depressions^43^. This high altitude and water availability enable a mosaic of vegetation, including more open areas as grassland and open scrubland, wet areas as gallery forests, until more dense areas with taller trees, as forest and wooded savanna^32^. In terms of geomorphology, CVNP is in the Brazilian Central Plateau and is formed by Araí Group, that includes the basement granite gneiss and contains quartzites, conglomerates, calcareous-pelitic rocks, sandstones, basalts, and siltstones; and also by Paranoá Group, which is above the Araí group and contains metasediments, including quartzites, metasiltstones, limestones and dolostones^43^.

### Tool sites and environmental sampling

**Figure 6:**
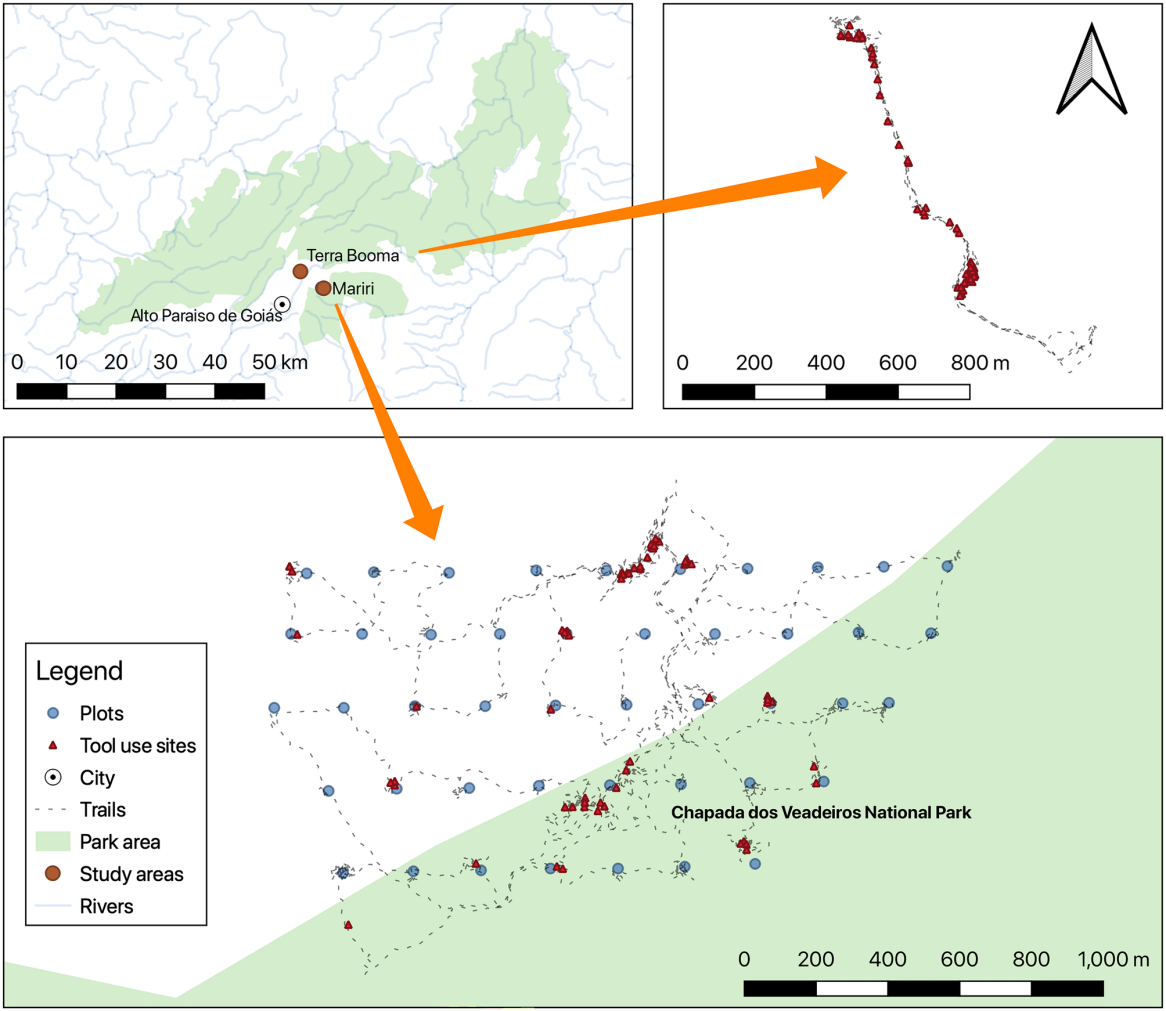
Map of trails and tool use sites (red triangles) recorded at Chapada dos Veadeiros National Park (CVNP), in two locations, Mariri (bottom) and Terra Booma (upper right). Plot sample locations at Mariri study site (bottom map, blue dots). Map created by Tiago Falótico, using QGIS 3.16 (https://www.qgis.org) and data of park area and hydrography from IBGE, Instituto Brasileiro de Geografia e Estatística (https://ibge.gov.br).

The stone tool use site mapping at CVNP was done following the same method of previous work^6^. For nine days (June 6–16, 2019), three researchers and a local field assistant walked 28.7 km of tracks on two locations at the south of the park (Mariri: − 14.10691, − 47.44084; Terra Booma: − 14.07716, − 47.48388; Fig. 6), actively looking for stone tool use processing sites. Both locations have at least one resident *S. libidinosus* group.

A “processing site” was characterized by the following items: (1) “anvil”, a flat surface used as a substrate for the processed encased food, (2) “hammer” on top or beside the anvil (within 1 m), and (3) remains of the processed encased food atop or adjacent (within 30 cm) to the anvil^44^.

When a processing site was identified, we recorded the following variables: GPS coordinates, anvil material and size (maximum length and width), stone tool material, weight and size (length, width, and thicknesses), and processed encased food. We used digital scales (to nearest 0.1 g), calipers (to nearest 0.1 mm), and measuring tapes (to nearest mm). We visually identified processed encased foods in each site and collected samples from nearby trees for botanical identification. When two resources were processed on the same anvil, we considered, for this analysis, the one that had more leftovers on and around the anvil. We collected, when possible, the remaining of processed resources at the anvils for the physical properties test (see below). However, most of the resources needed to be later collected fresh, to be in a similar stage to the one the monkeys process them, and thus were not associated with a cracking site, but they were collected opportunistically in nearby areas.

Samplings of processing sites and description of *S. libidinosus* tool use at ScaNP and SCoNP were done in previous studies^6,19^. Still, we revisited both locations in October/2019 to collect food samples for physical properties testing and plot samples (see below). We collected *Manihot*, *Annacardium,* and *Hymenaea* samples in the same areas of the previous works. At SCaNP, we collected additional behavioral data on tool use throughout two study periods with the Pedra Furada (PF) group (2018 and 2019). TF followed this group for a total of 242 h, recording all occurrences of tool use and measuring the tools used with the same protocol as above after the monkeys left. The PF group was also studied and described in previous studies in this area^19,20,23,24^. The data on the stone tools is available in Supplementary Table 1.

To characterize the availability of raw lithic material, we did plot samples at the three sites, in the living area of groups Pedra Furada (SCaNP), Gruta do Boi (SCoNP), and Mariri (CVNP). Forty-five plots of 10 × 10 m were done along 4.5 km transect lines, in 100 m intervals, covering the living area of at least one group of capuchin monkeys as part of a broader survey for ecological description. We identified stone tool use sites with the same methodology as above in each plot. To evaluate stone material and the amount available in the areas, we did a 50 × 50 cm subplot at the SW corner of each plot, where all stones with more than 3 cm (the lower size limit a capuchin monkey would use it as a tool^20^) were counted and classified by rock type, measured, and weighted.

### Food resources physical properties test

Mechanics were measured with a Lucas Scientific FLS-2 portable mechanical tester^45,46^. Although hardness is not a property per se, it is a derived concept traditionally used to measure the resistance to deforming under indentation. The technology of the equipment used can measure indentation of millimeter dimensions bringing more accurate results for such tests. The test used is called sharp indent (for more details, see^47^) and, in addition to the hardness values, gives us the elastic modulus, defined as the ratio of stress to strain in the elastic region^30^. Following an initial force loading, the displacement is held constant while recording the force decay for further 90 s or until the load stabilizes, forming a curve. Fitting a curve to the relaxation behavior allows the calculation of both an instantaneous (Ei) and infinite (E∞) elastic modulus. “Ei” represents the elastic modulus of a material if it could be loaded instantly, while “E∞” estimates the elastic behavior under an infinitely slow loading regime. In this study, we use “Ei” measurements, which are likely more relevant when investigating how food behaves under chewing loads^48^.

### Analyses

The resources’ hardness and elastic modulus values were compared using Kruskal–Wallis (KW) and Mann–Whitney U tests. Pairwise comparisons of values within and between sites were performed using post-hoc Mann–Whitney U tests with a Bonferroni correction for multiple comparisons. The effect size was calculated using Cohen’s d test (package “effsize”). Analyses were performed in R 4.2^49^ and the script is available in Supplementary Script 1.

### Ethical statement

The research regarding the monkeys was observational only and complied with protocols approved by the Animal Research Ethical Committee of the School of Arts, Sciences and Humanities, University of São Paulo (CEUA/EACH 002/2019); all methods were performed in accordance Brazilian law, under authorization from environmental agencies IBAMA/ICMBio (60134) and complied with the American Society of Primatologists Principles for the Ethical Treatment of Non‐Human Primates. The same authorization also gave permission to collect plants and seeds, and our study complies with national and University of São Paulo guidelines regarding experiments on plants.

## Supplementary Information

Supplementary Legends.

Supplementary Information.

Supplementary Table 1.

Supplementary Video 1.

## Data Availability

The data on stone tools and food physical properties analyzed during this study are included in this published article as supplementary information files. We also include the R script with the statistical tests performed. The remaining dataset is available from the corresponding author on reasonable request.
