# Supplementary material for: Stone tools differences across three capuchin monkey populations: food’s physical properties, ecology, and culture
Source: Sci Rep. 2022 Aug 23;12:14365. doi: 10.1038/s41598-022-18661-3 (PMC9399116; doi:10.1038/s41598-022-18661-3)
Supplement: Supplementary file 1 — Supplementary Legends. [file 41598_2022_18661_MOESM1_ESM.docx]

**Supplementary Material**

Supplementary Table 1. Data of the food resource physical properties measurements - Indentation Hardness (MPa) and Reduced Elastic Modulus (GPa) -, and stone tools weight (g), for capuchin monkeys’ population of Chapada dos Veadeiros National Park (CVNP), Serra da Capivara National Park (SCaNP) and Serra das Confusões National Park (SCoNP).

Supplementary Script 1. R script with the statistical tests performed.

Supplementary Video 1. Camera-trap footage of capuchin monkeys (*Sapajus libidinosus*) nut-cracking with stone tools at Chapada dos Veadeiros National Park, Brazil
